# Access to Alcohol: Geography and Prevention for Local Communities

**Published:** 1996

**Authors:** Paul J. Gruenewald, Alexander B. Millar, Peter Roeper

**Affiliations:** Paul J. Gruenewald, Ph.D., is senior research scientist, Alexander B. Millar, Ph.D., is senior research analyst, and Peter Roeper, Ph.D., is Community Trials Program manager at the Prevention Research Center, Pacific Institute for Research and Evaluation, Berkeley, California

**Keywords:** AOD availability, alcoholic beverage sales outlet, location and density of AOD outlets, societal AODR (alcohol and drug related) problems, violence, drinking and driving, traffic accident, geographical area, AOD prevention, intervention

## Abstract

Alcohol availability, measured in terms of geographic density of alcohol-sales outlets, is linked to specific patterns of alcohol-related motor vehicle crashes in communities. To curb alcohol-related problems such as violence, traffic crashes, and drinking and driving, community advocates often focus on reducing alcohol availability through modifications of zoning laws and licensing requirements. In developing interventions and policy activities to reduce alcohol availability, community policymakers should assess the effects of outlet density using an adequate community “biogeography” of the interrelationships among drinkers, their drinking environments, the locations of alcohol outlets, and the locations of alcohol problems. Communities also should consider that the economic development of downtown areas, which is often accompanied by the rapid growth of alcohol outlets, is likely to affect the incidence and prevalence of alcohol-related problems in surrounding areas.

Alcohol-related violence, traffic crashes and injury, and drinking and driving are all issues of concern to communities. To curb alcohol-related problems such as these, community advocates frequently focus on reducing alcohol availability, a policy area typically under local control (e.g., through zoning laws and licensing requirements). Restrictions on alcohol availability may in turn, however, encounter resistance from economic development and other local community interests. The challenge to communities is to balance these clashing interests, whereas the challenge to researchers is to provide scientific evidence to inform these important policy decisions.

This article presents recent research findings on the relationship between the availability of alcohol within local communities and the incidence and prevalence of alcohol-associated problems, most notably violence and drinking and driving. In the studies mentioned, the geographical areas investigated range from collections of small cities (Scribner et al. 1994, 1995) and census tracts within cities (Parker and Cartmill 1996; Alaniz et al. 1996) to areas consisting of only several city blocks (Gruenewald et al. 1996). Within these communities, alcohol’s availability may be determined by a number of factors that often relate to the relative ease or difficulty of obtaining it (Gruenewald et al. 1993, 1996). These factors include the days and hours when outlets are licensed to sell alcohol, the location of outlets (i.e., proximity to highways, schools, colleges, and so forth), and the number of alcohol outlets per kilometer of roadway (i.e., geographic availability) (Gruenewald 1993). Alcohol outlets may be State controlled or privately owned businesses, and alcohol may be consumed on the premises (e.g., at bars and restaurants) or purchased for later consumption off the premises (e.g., at grocery, convenience, or liquor stores). These two types of outlets are known as on-premises and off-premises establishments.

## The Geography of Availability and Alcohol-Related Problems

Most recent studies conclude that a relationship exists among alcohol availability, alcohol use, and alcohol-related problems (Gruenewald 1993). One recent study found that a ban on Sunday alcohol sales in a Georgia community appeared to reduce the incidence of drinking and driving on all days (Ligon and Thyer 1993). A study of changes over time in alcohol sales, the incidence of crime, and drinkers’ involvement in the criminal justice system in England and Wales suggested that easy access to alcohol may be implicated in a wide range of crimes (Ensor and Godfrey 1993). In another study, the elimination of a prohibition against wine sales in New Zealand grocery stores was found to increase such sales (Wagenaar and Langley 1995). Likewise, the elimination of retail wine monopolies in five States was found to increase wine sales (Wagenaar and Holder 1995). Given the strong relationship found between alcohol sales and problems such as alcohol-related crashes (Gruenewald and Ponicki 1995*a*), cirrhosis mortality (Gruenewald and Ponicki 1995*b*), and suicide (Gruenewald et al. 1995), increases in alcohol sales may be related to considerable increases in these problems.

Scribner and colleagues (1994, 1995) provided the first evidence suggesting statistically reliable associations at the local level between the number of alcohol outlets (i.e., outlet density) and the frequency of motor vehicle crashes and rates of violent assaults. Although their studies had a number of methodological and statistical problems,^1^ the researchers did demonstrate, based on data they collected in 72 contiguous cities in the Los Angeles basin, the feasibility of performing extensive community-based geographic analyses of alcohol availability and associated problems. Scribner and colleagues demonstrated significant positive relationships between measures of outlet density and alcohol-involved traffic crashes and rates of violent assault. Although motor vehicle crashes primarily were associated with drinking in on-premises establishments, rates of violent assaults were related to greater densities of both on- and off-premises establishments.

Parker and Cartmill (1996) also demonstrated statistically significant links between geographic availability and rates of violent assault across census tracts in a small California community. These authors found that rates of violent assault were related positively to the density of off-premises outlets. Alaniz and colleagues (1996) generalized this work to several cities, demonstrating an association between youthful violence and the geographic availability of alcohol. Each of these studies presumed the existence of a geographic link between the availability of alcohol and alcohol problems (e.g., that individual drinkers purchased or consumed alcohol in specific locations, then drove elsewhere and subsequently were involved in drinking-related crashes), and each presented data largely supporting the validity of these presumptions.

In a study of the connections between alcohol sales and the prevalence of drinking and driving in Perth, Australia, Gruenewald and colleagues (in press) demonstrated that incidence of drinking and driving (both involving and not involving crashes) could be predicted by the type of establishment that offered alcohol for sale and the volume of alcohol sold to the driver. Specifically, outlets that sold the greatest quantity of alcohol, particularly nightclubs serving beer and spirits, were likely to produce the largest proportion of drinking drivers.

### A Closer Look at the Geography of Alcohol Problems

This research has established that the availability of alcohol is linked to specific patterns of alcohol-related motor vehicle crashes in community settings. To better clarify the link between the number of alcoholic beverage outlets and the incidence of alcohol-related crashes in four California communities, Gruenewald and colleagues (1996) studied the relationships between traffic crashes and several other geographic factors, including environmental, demographic, and drinking variables and measures of outlet density.

The researchers selected traffic-crash measures that represented specific alcohol-related events (e.g., single-vehicle nighttime [SVN] crashes, which are most likely to involve alcohol) (Holder et al. 1995). Environmental variables were those that described the general ecological features related to traffic patterns in the communities (e.g., average daily traffic flow, cross-street counts, and total number of intersections). Demographic and drinking variables were obtained from a general population survey of adults and described population characteristics and factors relevant to drinking (e.g., income, average age, frequency of drinking, and average drinking levels). Measures of outlet density represented the physical availability of alcohol by outlet type measured over a metric scale representing use of space (i.e., the number of bars, restaurants, grocery, and liquor stores per kilometer of roadway). Using specialized statistical techniques (i.e., geostatistical regression models) to correct for problems that arise in the analysis of geographic data, the researchers found that environmental, demographic, drinking-behavior, and outlet-density variables were related to crash rates across all the geographic units in the study.

**Figure 1:**
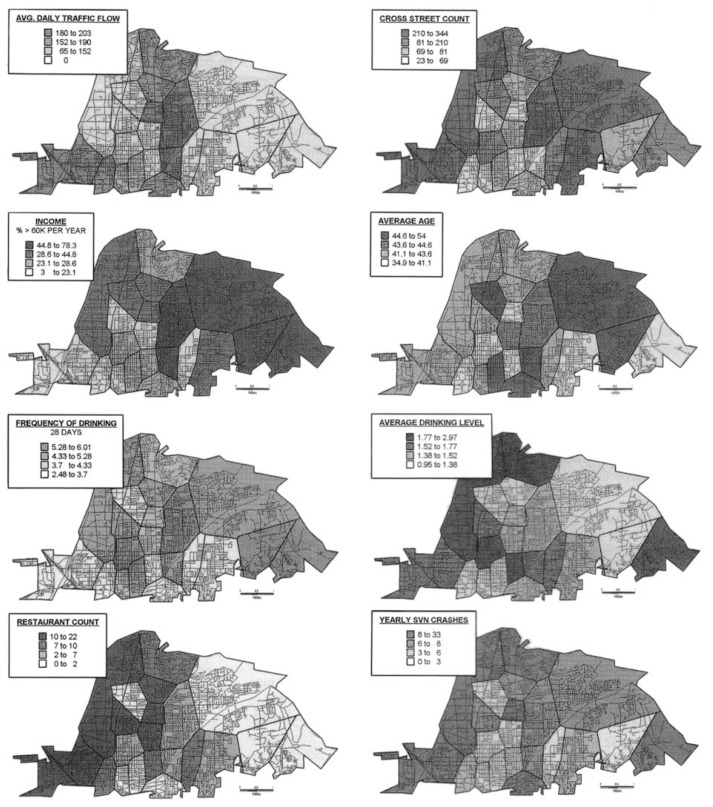
Geographic distributions of environmental and demographic measures for one California community. The environmental measures represent general features of the community related to traffic crashes (e.g., average daily traffic flow and number of cross streets). Demographic and drinking measures are characteristic of the population (e.g., frequency of drinking, average drinking level, income, and average age). Restaurant count represents the physical availability of alcohol. Traffic-crash measures refer to specific alcohol-related events (e.g., single-vehicle nighttime [SVN] crashes). As shown in these maps, substantial geographic variation exists within the community for all measures.

To ensure accurate comparison, each community was partitioned into a number of relatively small geographic units whose sizes generally reflected local population densities (i.e., the units were smallest in the most dense areas). The ranges of various measures in the study across these geographic units differed from one unit to another, sometimes dramatically. For example, the proportion of people who abstained from using alcohol ranged from 3 to 61 percent, unemployment rates varied from 0 to 12 percent, and outlet densities varied from 0 to 4 outlets per kilometer of roadway. (See figure 1 for examples of geographic distribution maps generated for one of the four communities.)

**Figure 2:**
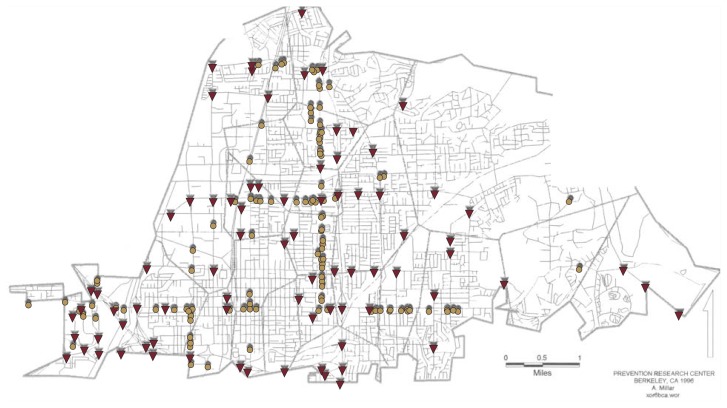
Mapping the relationship of restaurant availability to single-vehicle nighttime (SVN) crashes. The street map of each local community is used to identify outlet locations (e.g., tan dots represent restaurants) and SVN crashes (represented by red triangles) within geographic units. These data demonstrate a significant relationship between restaurant densities and SVN crashes both across and within the communities.

The street map of each local community then was used to identify outlet locations and SVN crashes within each geographic unit. By mapping both of these variables onto the geographic units, annual rates of SVN crashes could be related to locations of outlets within and between community study areas (figure 2).

The results of these data analyses demonstrated significant relationships between restaurant densities and SVN crashes both across and within the communities. Specifically, the greater the level of alcohol availability through restaurants, the higher the rates of alcohol-related crashes across the geographic units of the study. Overall, 10 percent greater restaurant density was related to 1.7 percent higher crash rates. The effect of restaurant density on crash rates extended to adjacent areas as well: Rates of alcohol-related crashes within each study area were affected by restaurant density both within those areas and in surrounding areas, demonstrating the “spreading” effects of alcohol availability in communities. Interestingly, the positive relationship between outlet density and traffic crashes in these communities was found primarily for restaurants. No significant effects on traffic crash rates were found for increased densities of bars or off-premises alcohol outlets (Gruenewald et al. 1996). Restaurants appear to supply the greatest proportion of drinking drivers, at least in California, where restaurants are frequented about twice as often as bars for the consumption of alcohol.

**Table:** Timeline for Intervention Activity for Three Experimental Communities in the Community Trials Project

Year	Quarter	Experimental Communities		

		Community 1	Community 2	Community 3
1 (1992–1993)	1–2	Begin coalition development and scientific support for project	Begin coalition development and scientific support for project	Begin coalition development and scientific support for project
	3–4	Begin active phase of coalition-based implementation of scientifically supported intervention acitivities	Begin active phase of coalition-based implementation of scientifically supported intervention acitivities	Begin active phase of coalition-based implementation of scientifically supported intervention acitivities

2	1	Ban on alcohol use in city parts CUP regulations maintained in “alcohol overlay zone” (east side of city) Request to city council to form Availability Task Force	Request to city council to form Access Task Force	
	2	City adopts new regulations on alcohol in farm labor camps Hispanic chamber of commerce bans alcohol sales at sponsored event One CUP approved for offsite outlet First meeting of Availability Task Force		County Coalition Task Force on Access formed and first meeting takes place
	3			
	4	Commnity training on regulating access to alcohol Alcohol advertising and selling restrictions increased on city property	First meeting of Access Task Force	

3 (1994–1995)	1	One CUP denied for offsite outlet		
	2	Restriction on alcohol availability increased at summer festival	One off-sale outlet has license challenged Nearby city bans alcohol on public beaches City considers easing alcohol laws to promote downtown growth	
	3	Planning commission holds public eharing on new alcohol CUP ordinance One CUP revoked for onsite outlet	Nearby city bans alcohol at public sporting events	
	1–2	Grant awarded to sheriff to oversee bar and restaurant sales in “parent” county CUP process for alcohol outlets extended citywide CUP requiring responsible beverage service training for new licenses	Access Task Force completes first draft recommendations for CUP regulations	Extraterritorial jurisdiction ordinance introduced in city council (allows city zoning to extend up to 3 miles beyond city limits)

4 (1995–1996)	1		Public presentation of recommended CUP ordinances warmly received	New city ordinance imposed requiring minimum distance between bars and restaurants Citizens successfully oppose issuance of new on-site alcohol license City council agrees to testify in alcohol license hearings
	2	Downtown exempted from selected CUP requirements	Chamber of commerce takes position against new CUP ordinances	In response to citizen requests, State ABC agrees to conduct license hearings in local community instead of State capitol
	3		Area city passes new CUP inceasing distances between off-sale outlets and schools and residential areas	
	4		CUP draft presented to city planning, police, and redevelopment departments; includes new distance requirements Neighbor city passes comprehensive CUP City council considers easing alcohol laws to promote downtown growth	Citizens oppose issuance of new license—license approved, but with additional conditions

5 (1996)	1	Neighborhood groups trained to monitor alcohol outlets Planning department recommends enhanced CUP ordinance to city council	Minimum military drinking age raised to 21	

NOTE: CUP = Conditional Use Permit (i.e., a permit for sales of alcohol conditional on restrictions established by city or county governments).

If restaurants are the type of establishments that produce the most drinking drivers, restaurant density could be an important predictor of community patterns of alcohol-related motor vehicle crashes. The distribution of restaurants in a community would predict the places where alcohol-related crashes would be likely to occur. Thus, research on alcohol availability is of considerable importance from a community perspective because it demonstrates the specific geographic effects of drinking on motor vehicle crashes across communities. The current research demonstrates the degree to which neighborhoods differ in both demographic composition and alcohol use, as well as the extent to which geographically specific effects of availability on alcohol-related problems are to be expected.

## Putting Research Into Practice: Three Case Studies

A recent community trial to test the efficacy of preventive interventions (Holder et al. 1995) showed the degree to which community responses to reducing alcohol availability and alcohol-related problems can vary across community settings. The Community Trials study included five preventive interventions intended to decrease the rates of alcohol-related trauma resulting from both traffic crashes and other causes in three experimental communities. (For more information on the Community Trials, see the article by Holder, pp. 252–260, and the box on p. 257.) One of the five interventions, the access intervention, aimed to reduce the physical availability of alcohol by curtailing the activities of alcohol outlets through changes in hours or days of sale, closing “problem” outlets (e.g., outlets with a previous history of illegal alcohol sales), restricting the opening of new outlets, or initiating planning and zoning regulations designed to decrease outlet density. These access-related activities in the three communities (summarized in table) pitted the health interests of the community against the apparent economic interests of community developers and received many diverse reactions from the parties involved.

As noted by Gruenewald and Millar (1996), although the three communities had much in common, each one implemented the access intervention in slightly different ways. The first community rapidly developed the intervention, moving quickly to initiate bans on alcohol use in city parks, maintain current planning and zoning restrictions on alcohol outlets, and develop new planning and zoning regulations. These efforts were accompanied by restrictions on alcohol availability at public events, continued protests over problem outlets, and preparation of a model Conditional Use Permit (CUP) ordinance (i.e., a permit for alcohol sales that is conditional on restrictions established by city or county governments). Nonetheless, during the intervention period (1995), the city council exempted the downtown area from selected CUP requirements, enabling the rapid growth of outlets for the purpose of community development.

The second community moved somewhat more slowly in developing the access intervention, receiving support from the city council to form an access task force and suggesting revisions to alcohol-related ordinances. However, rather early in the intervention period (1994), the city council had begun considering easing alcohol regulations to promote downtown growth, a move supported by the local chamber of commerce, which ultimately opposed all new CUP ordinances.

The third community was working within the context of a comparatively primitive regulatory environment. For example, until midway into the intervention period (1995), alcohol licenses were reviewed in the State capitol, effectively blocking local representation at licensing hearings. When the State Alcoholic Beverage Control agreed to conduct licensing hearings in the local community, the regulatory change allowed local citizens to intervene more effectively in the licensing process. Around the same time, the city council began to allow local police to testify in alcohol licensing hearings. The police provided official information concerning problem outlets to help lobby for their closing.

Although the three communities’ approaches to reducing alcohol-related problems diverged, each community began the process in the same way: by developing a coalition and gathering scientific support for the intervention activities. These three brief case studies show that local communities can apply the scientific evidence supporting the existence of a relationship between alcohol availability and alcohol problems. As a prime example, in all three communities, advocates for constraints on alcohol availability used evidence from studies such as those cited in this article to suggest that increasing the required minimum distance between outlets would be the best first step toward reducing availability and decreasing alcohol-related problems. Thus, by proposing planning and zoning changes, the advocates implemented the scientific observation that geographic outlet density has the greatest impact on alcohol-related problems in the community.

## Toward a Biogeography of Drinking Problems

From the perspective of those interested in reducing alcohol problems in their communities, the physical availability of alcohol makes an attractive policy target. Unlike alcoholic beverage taxes, the location of alcohol outlets is subject to local regulation and can be closely monitored by community groups. In addition, and again unlike alcoholic beverage taxes, alterations in the physical availability of alcohol can have immediate effects on patterns of alcohol use within communities and can reduce access substantially in locations where high-risk behavior is likely (e.g., in bars and restaurants near highways). Another appeal of regulating alcohol availability is that regulations affect access to all beverages sold by an outlet (regardless of price) to all segments of the population (regardless of income). In contrast, alcohol taxation takes place in an environment where beverage prices differ by orders of magnitude and the effects of a tax may be diluted by the range of beverage prices and other factors. (For more information on alcohol tax policy, see the article by Kenkel and Manning, pp. 230–238.)

To support community activities in reducing the availability of alcohol, much further research must be performed. As noted in this article, the evidence for a direct link between availability, alcohol use, and specific alcohol-related problems at the community level has been scant, consisting of only a handful of studies. In addition, theoretical models of the geographic relationships between availability and alcohol-related problems are still in their infancy (see, for example, Gruenewald et al. 1996). Although the demography and drinking behavior of local populations may differ dramatically across geographic areas, no theoretical or empirical study exists that has examined how these geographic differences relate to alcohol problems. Researchers undertaking such studies seek to answer many questions: Are there pools of high-risk populations on which outlets draw that produce alcohol-related problems? Are there areas in which the conjunction of high outlet density and high-risk populations is particularly likely to produce problems? How can the relationships between pools of alcohol users and sources of alcohol be geographically portrayed? What is an adequate theory of the processes by which changes in alcohol use and alcohol-related problems occur in response to alterations in availability? To the extent that these questions can be satisfactorily addressed, communities will be able to respond to local alcohol problems.

To support the rational development of community interventions and policy activities intended to reduce the potentially harmful consequences of alcohol availability, the construction of an adequate “biogeography” of alcohol problems at the community level is needed. A biogeography of alcohol problems would describe the interrelationships among drinkers, their drinking environments, the locations of alcohol outlets, and evidence of alcohol problems. For example, in cases of alcohol dependence and heavy or binge drinking, the convergence of populations at risk (e.g., young to middle-aged single males) and increased access to alcohol may be key to determining the geographic distribution of types of drinking problems. In the case of drinking and driving, the location of alcohol use (e.g., restaurants)—not the location of the drinker’s home—may be directly related to motor vehicle crash sites (Gruenewald et al. 1996). As another example, the combination of a youthful pool of potential drinkers and greater off-premises alcohol availability may determine rates of under-age drinking and problems related to such use.

## Conclusion

Many communities are confronted with serious problems regarding alcohol availability and associated alcohol-related problems. Moreover, drinking and driving and the crashes that result are not limited to those areas of the community with the greatest concentration of alcohol outlets; the effects spread across adjacent community areas that may have comparatively low alcohol-outlet densities. Therefore, communities need to assess the impact of increased outlet density in broader geographic terms. In addition, communities must consider that the economic development of downtown areas, often accompanied by the rapid growth of alcohol outlets, is likely to be accompanied by rapid growth of alcohol problems in surrounding areas.

In communities grappling with alcohol-related problems, activists will continue to pose the following central questions: “How do we prevent or reduce alcohol-related problems in the community?” and “Are reductions in availability sufficient to reduce these alcohol problems, or are changes in enforcement and preventive education also necessary?” The advancement of theoretical models of the geography of alcohol problems and the application of methods to mapping these problems will help answer these questions and make possible more targeted preventive interventions.

## Footnotes

1 Scribner and colleagues measured alcohol availability in terms of outlets per person. However, measurements in terms of outlets per geographic unit better reflect a consumer’s ease or difficulty in obtaining alcohol. Analyses of fixed geographic units usually are interrelated (i.e., have a great deal of spatial dependence) and thus require specialized statistical techniques for analysis.
